# Graph theory applications in congenital heart disease

**DOI:** 10.1038/s41598-023-38233-3

**Published:** 2023-07-10

**Authors:** Yao-Ting Lee, Shyh-Jye Chen

**Affiliations:** Department of Medical Imaging, National Taiwan University Hospital and Children Hospital, National Taiwan University, 7 Chung-Shan South Road, Taipei, 10002 Taiwan

**Keywords:** Congenital heart defects, Applied mathematics

## Abstract

Graph theory can be used to address problems with complex network structures. Congenital heart diseases (CHDs) involve complex abnormal connections between chambers, vessels, and organs. We proposed a new method to represent CHDs based on graph theory, wherein vertices were defined as the spaces through which blood flows and edges were defined by the blood flow between the spaces and direction of the blood flow. The CHDs of tetralogy of Fallot (TOF) and transposition of the great arteries (TGA) were selected as examples for constructing directed graphs and binary adjacency matrices. Patients with totally repaired TOF, surgically corrected d-TGA, and Fontan circulation undergoing four-dimensional (4D) flow magnetic resonance imaging (MRI) were included as examples for constructing the weighted adjacency matrices. The directed graphs and binary adjacency matrices of the normal heart, extreme TOF undergoing a right modified Blalock–Taussig shunt, and d-TGA with a ventricular septal defect were constructed. The weighted adjacency matrix of totally repaired TOF was constructed using the peak velocities obtained from 4D flow MRI. The developed method is promising for representing CHDs and may be helpful in developing artificial intelligence and conducting future research on CHD.

## Introduction

Congenital heart diseases (CHDs) often involve abnormal connections between chambers, vessels, and organs, such as atrioventricular discordance, ventriculoarterial discordance, and cardiovascular shunts^1^. Management of CHDs may be related to the creation or elimination of the connections^2,3^. Graph theory is a branch of mathematics that can be applied to map complex networks between objects, and it is widely used in biomedical sciences^4^. Some studies have applied graph theory for the analysis of the human heart networks and have proposed its medical applications^5–10^. However, these studies have mainly focused on mathematical theories rather than on the pathophysiology of the heart. Moreover, the specific diseases and structural anomalies of the heart have not been considered. Therefore, this study developed a new method to represent CHDs based on graph theory.

## Methods

Due to the retrospective nature of this study, the need of informed consent was waived by the National Taiwan University Hospital Research Ethics Committee. The study protocol strictly adheres to the ethical guidelines of the 1975 Declaration of Helsinki and was conducted in at a single tertiary referral hospital.

The definitions and terminologies used in graph theory are based on those in previous studies^4,6^. We denote the directed graph of the heart by *G*(*V, E*), where *V* is a set of vertices, and *E* is a set of edges between the vertices. Vertices are defined as the spaces through which blood flows and are numbered in order. Let *V* = {*v*_1_*, v*_2_*,…, v*_*i*_*,…*}; the vertices of the directed graph of the normal heart are assigned as follows: *v*_1_ = superior vena cava (SVC), *v*_2_ = inferior vena cava (IVC), *v*_3_ = right atrium (RA), *v*_4_ = right ventricle (RV), *v*_5_ = pulmonary trunk (PT), *v*_6_ = right pulmonary artery (RPA), *v*_7_ = left pulmonary artery (LPA), *v*_8_ = right lung (RL), *v*_9_ = left lung (LL), *v*_10_ = right upper pulmonary vein (RUPV), *v*_11_ = right lower pulmonary vein (RLPV), *v*_12_ = left upper pulmonary vein (LUPV), *v*_13_ = left lower pulmonary vein (LLPV), *v*_14_ = left atrium (LA), *v*_15_ = left ventricle (LV), *v*_16_ = ascending aorta (AAo), *v*_17_ = aortic arch (AoA), *v*_18_ = descending aorta (DAo), *v*_19_ = innominate artery (IA), *v*_20_ = left common carotid artery (LCCA), *v*_21_ = left subclavian artery (LSCA), *v*_22_ = right common carotid artery (RCCA), *v*_23_ = right subclavian artery (RSCA), and *v*_24_ = systemic circulation target organs (SCTO). We use vertex *v*_24_ to represent all organs involved in the systemic circulation, such as the brain, visceral organs, and soft tissues. Edges are defined as the blood flow between the spaces. If blood flows between two spaces, then an edge is formed between the two vertices. The direction of the edge is the direction of the blood flow. For example, if the blood flows from RA to RV, an edge is formed between *v*_3_ and *v*_4_ in the direction from *v*_3_ to *v*_4_.

Tetralogy of Fallot (TOF) is the most common cyanotic CHD, and patients with TOF sometimes require shunt creation, especially in the case of extreme TOF, which is characterized by pulmonary atresia^2,11,12^. Transposition of the great arteries (TGA) is a typical example of CHD with abnormal connections caused by ventriculoarterial discordance^13^. The most common form of TGA is d-TGA. Isolated d-TGA is incompatible with life without the presence of a shunt for blood mixing, and the most common shunt in d-TGA is a ventricular septal defect (VSD)^14^. Hence, we selected cases of extreme TOF managed using a right modified Blalock–Taussig (mBT) shunt and of d-TGA with a VSD as examples for constructing directed graphs.

We constructed adjacency matrices of the directed graph of the heart, including binary (unweighted) and weighted adjacency matrices. Let **A** = [*a*_*ij*_] be a binary adjacency matrix of a graph *G*(*V, E*); *a*_*ij*_ equals “1” if there is an edge from *v*_*i*_ to *v*_*j*_ and “0” otherwise. For the heart, the connectivity weights can denote hemodynamic parameters such as flow, peak velocity, and pressure gradient. Echocardiography and cardiac magnetic resonance imaging (MRI) are common imaging modalities used to measure the aforementioned parameters^15,16^. In this study, we used four-dimensional (4D) flow MRI to construct the weighted adjacency matrices because the technique enables retrospective measurements of blood flows at arbitrary cross-sectional planes of a given vessel^17^. Patients with totally repaired TOF, surgically corrected d-TGA, and Fontan circulation undergoing 4D flow MRI were included as examples. Peak velocities of blood flows, which can be used to detect valvular, vascular, and anastomotic stenosis, were measured as weights in the weighted adjacency matrices by using 4D flow MRI^18^. Pulmonary stenosis is common in cases of totally repaired TOF and surgically corrected d-TGA. Hence, the peak velocities were measured between the RV, PT, RPA, and LPA. For the Fontan circulation, the peak velocities were measured between the SVC, RPA, IVC, and LPA to detect the potential anastomotic stenosis and increased pulmonary pressure.

## Results

### Directed graphs of the heart

**Figure 1:**
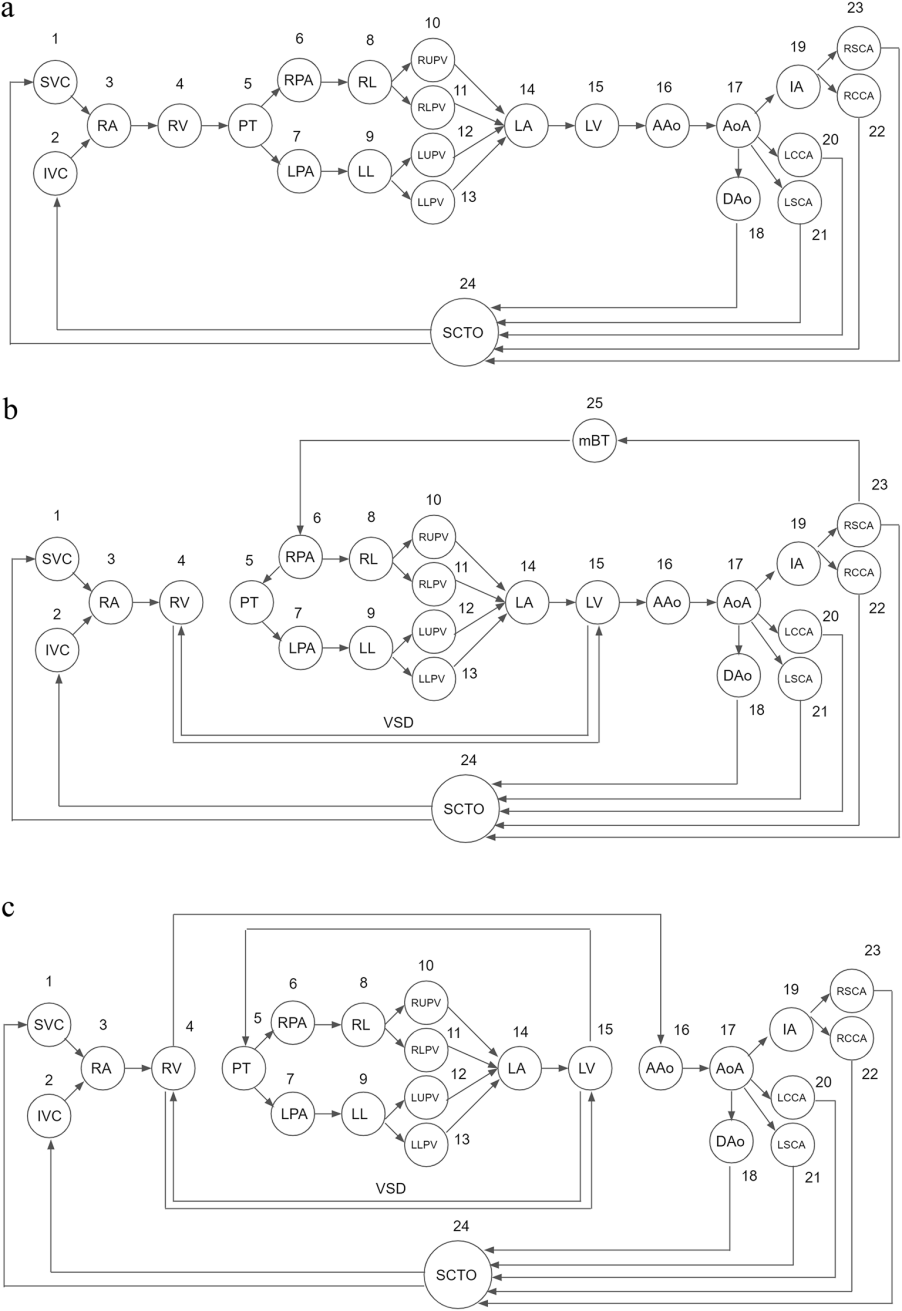
Directed graphs of (**a**) the normal heart, (**b**) extreme TOF undergoing a right mBT shunt, and (**c**) d-TGA with a VSD. In the case of extreme TOF undergoing a right mBT shunt, the edge from the RV to PT is absent due to pulmonary atresia. The bidirectional edges between the RV and LV represent the VSD. An additional vertex, *v*_25_, represents the mBT shunt, which allows blood flow from the RSCA to the RPA to supply the lungs. The direction of the edge between PT and RPA was reversed due to the presence of pulmonary atresia and the blood flow supplied by the right mBT shunt. In the graph of d-TGA, the connections between the ventricles and great arteries are discordant (RV to AAo, LV to PT). A VSD forms the paths from systemic venous return (SVC, IVC) to the lungs (RL, LL) and the paths from the lungs to the arterial supply (AAo). This allows patients with d-TGA to return deoxygenated blood from the body, exchange gases in the lungs, and pump oxygenated blood to the systemic circulation. *AAo* ascending aorta, *IVC* inferior vena cava, *LL* left lung, *LPA* left pulmonary artery, *LV* left ventricle, *mBT shunt* modified Blalock-Taussig shunt, *PT* pulmonary trunk, *RL* right lung, *RPA* right pulmonary artery, *RSCA* right subclavian artery, *RV* right ventricle, *SVC* superior vena cava, *TGA* transposition of the great arteries, *TOF* tetralogy of Fallot, *VSD* ventricular septal defect.

The directed graphs of the normal heart, extreme TOF undergoing a right mBT shunt, and d-TGA with a VSD are shown in Fig. 1. First, we drew the directed graph of the normal heart. Second, in the case of extreme TOF undergoing a right mBT shunt, the connection between the RV and PT was removed because of pulmonary atresia. An edge from the RV to LV and an edge from the LV to RV were added to the graph of the normal heart to represent VSD. Furthermore, the right mBT shunt was added to the graph as an additional vertex *v*_25_ = mBT shunt, which allowed blood flow from the RSCA to RPA first, and then to supply the bilateral lungs. The direction of the edge between PT and RPA was reversed due to the presence of pulmonary atresia and the blood flow supplied by the right mBT shunt. Finally, we drew the graph of d-TGA by eliminating the normal connections between the ventricles and great arteries (RV to PT, LV to AAo) and adding discordant connections (RV to AAo, LV to PT) to the graph of the normal heart. A VSD is mandatory to form the paths from systemic venous return (SVC, IVC) to the lungs (RL, LL) and the paths from the lungs to the arterial supply (AAo). This allows patients with d-TGA to return deoxygenated blood from the body, exchange gases in the lungs, and pump oxygenated blood to the systemic circulation that keeps survival.

### Binary adjacency matrices of the hearts

**Figure 2:**
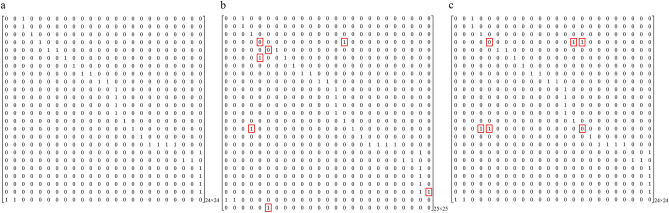
Binary adjacency matrices of (**a**) the normal heart, (**b**) extreme TOF undergoing a right mBT shunt, and (**c**) d-TGA with a VSD. The values in the red boxes denote the differences between the binary adjacency matrices of the normal heart and CHDs. Let **B** = [*b*_*ij*_] and **C** = [*c*_*ij*_] be the binary adjacency matrices of extreme TOF undergoing a right mBT shunt and d-TGA with a VSD, respectively. For the matrix of extreme TOF undergoing a right mBT shunt, an additional vertex caused the dimension of the matrix to increase from 24 × 24 to 25 × 25. The elements *b*_23,25_ = 1 and *b*_25,6_ = 1 represent the blood flow from RSCA to RPA via the right mBT shunt. The elements *b*_4,15_ = 1 and *b*_15,4_ = 1 represent the bidirectional VSD. The element *b*_4,5_ = 0 indicates pulmonary atresia. The elements *b*_5,6_ = 0 and *b*_6,5_ = 1 indicate the reversed blood flow from the RPA to the PT. For the matrix of d-TGA with a VSD, the elements *c*_4,5_ = 0 and *c*_15,16_ = 0 represent disconnection between the RV and PT, as well as between the LV and AAo. The elements *c*_4,16_ = 1 and *c*_15,5_ = 1 represent abnormal connections between the RV and AAo, as well as the LV and PT. The matrix of d-TGA can also be considered as the result of interchanging the 5th column (PT) and the 16th column (AAo) of the matrix of the normal heart. *AAo* ascending aorta, *CHD* congenital heart disease, *LV* left ventricle, *mBT shunt* modified Blalock-Taussig shunt, *PT* pulmonary trunk, *RPA* right pulmonary artery, *RSCA* right subclavian artery, *RV* right ventricle, *TGA* transposition of the great arteries, *TOF* tetralogy of Fallot, *VSD* ventricular septal defect.

The binary adjacency matrices of the normal heart, extreme TOF undergoing a right mBT shunt, and d-TGA with a VSD are shown in Fig. 2. Let **B** = [*b*_*ij*_] and **C** = [*c*_*ij*_] be the binary adjacency matrices of extreme TOF undergoing a right mBT shunt and d-TGA with a VSD, respectively. For the matrix of extreme TOF undergoing a right mBT shunt, an additional vertex caused the dimension of the matrix to increase from 24 × 24 to 25 × 25. The elements *b*_23,25_ = 1 and *b*_25,6_ = 1 represent the blood flow from RSCA to RPA via the right mBT shunt. The elements *b*_4,15_ = 1 and *b*_15,4_ = 1 represent the bidirectional VSD. The element *b*_4,5_ = 0 indicates pulmonary atresia. The elements *b*_5,6_ = 0 and *b*_6,5_ = 1 indicate the reversed blood flow from the RPA to the PT. For the matrix of d-TGA with a VSD, the elements *c*_4,5_ = 0 and *c*_15,16_ = 0 represent disconnection between the RV and PT, as well as between the LV and AAo, respectively. The elements *c*_4,16_ = 1 and *c*_15,5_ = 1 represent abnormal connections between the RV and AAo, as well as the LV and PT. In summary, these elements indicate ventriculoarterial discordance. The matrix of d-TGA can also be considered as the result of interchanging the 5th column (PT) and the 16th column (AAo) of the matrix of the normal heart.

### Weighted adjacency matrices of the hearts

**Figure 3:**
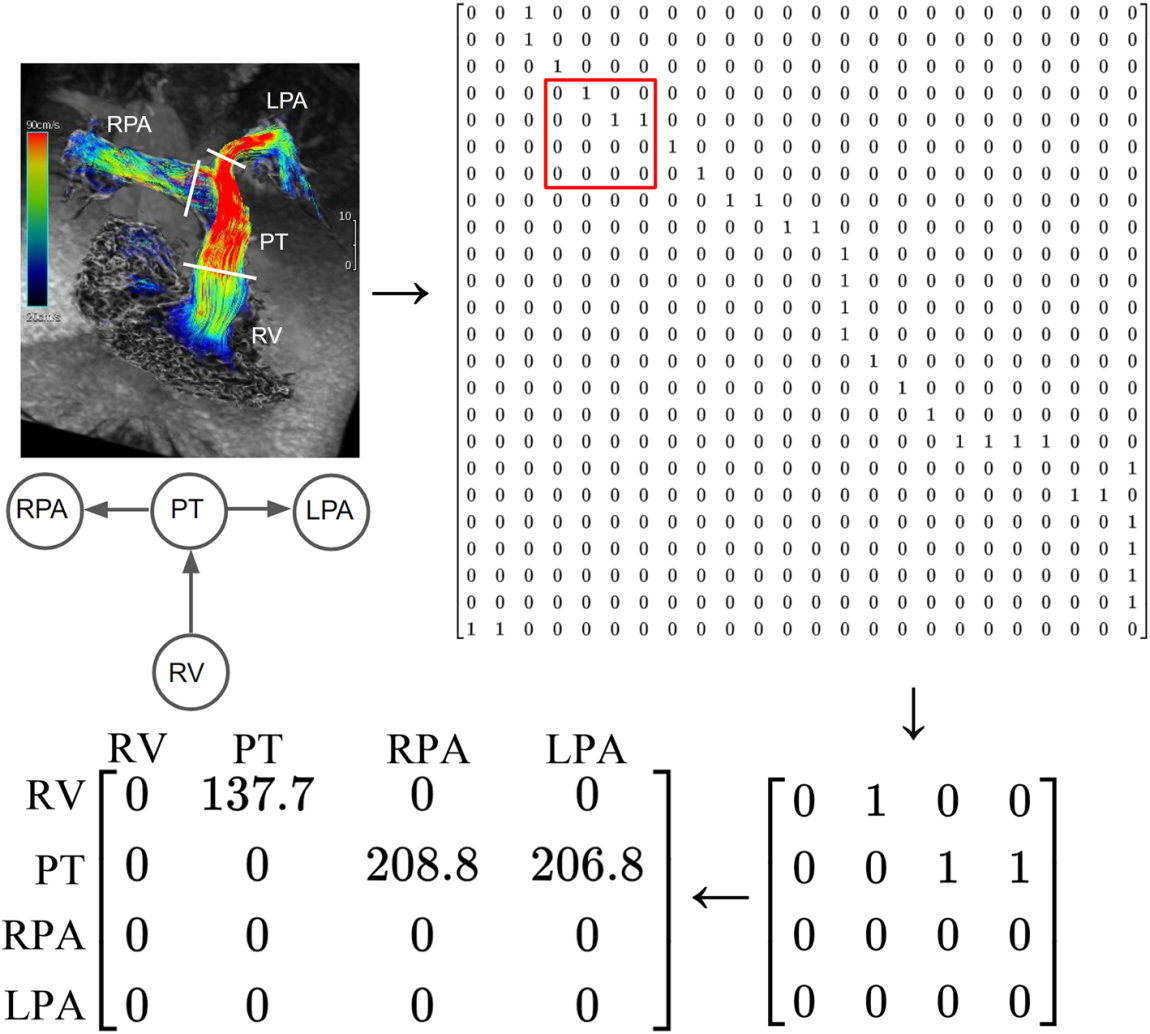
Construction of the weighted adjacency matrix based on the results of 4D flow MRI. Blood flow visualization with velocity color-coded streamlines on 4D flow MRI. In the case of totally repaired TOF, we were interested in the peak velocities of blood flows between RV, PT, RPA, and LPA. The peak velocities (in cm/s) were measured at the boundaries of the spaces on 4D flow MRI (pulmonary valve and the origins of the RPA and LPA, the short white lines). The submatrix including the vertices of interest was derived from the binary adjacency matrix of totally repaired TOF (red box). After the weights and corresponding elements of the submatrix were combined, the weighted adjacency matrix of peak velocity was obtained. *4D flow MRI* four-dimensional flow magnetic resonance imaging, *LPA* left pulmonary artery, *PT* pulmonary trunk, *RPA* right pulmonary artery, *RV* right ventricle, *TOF* tetralogy of Fallot.

**Figure 4:**
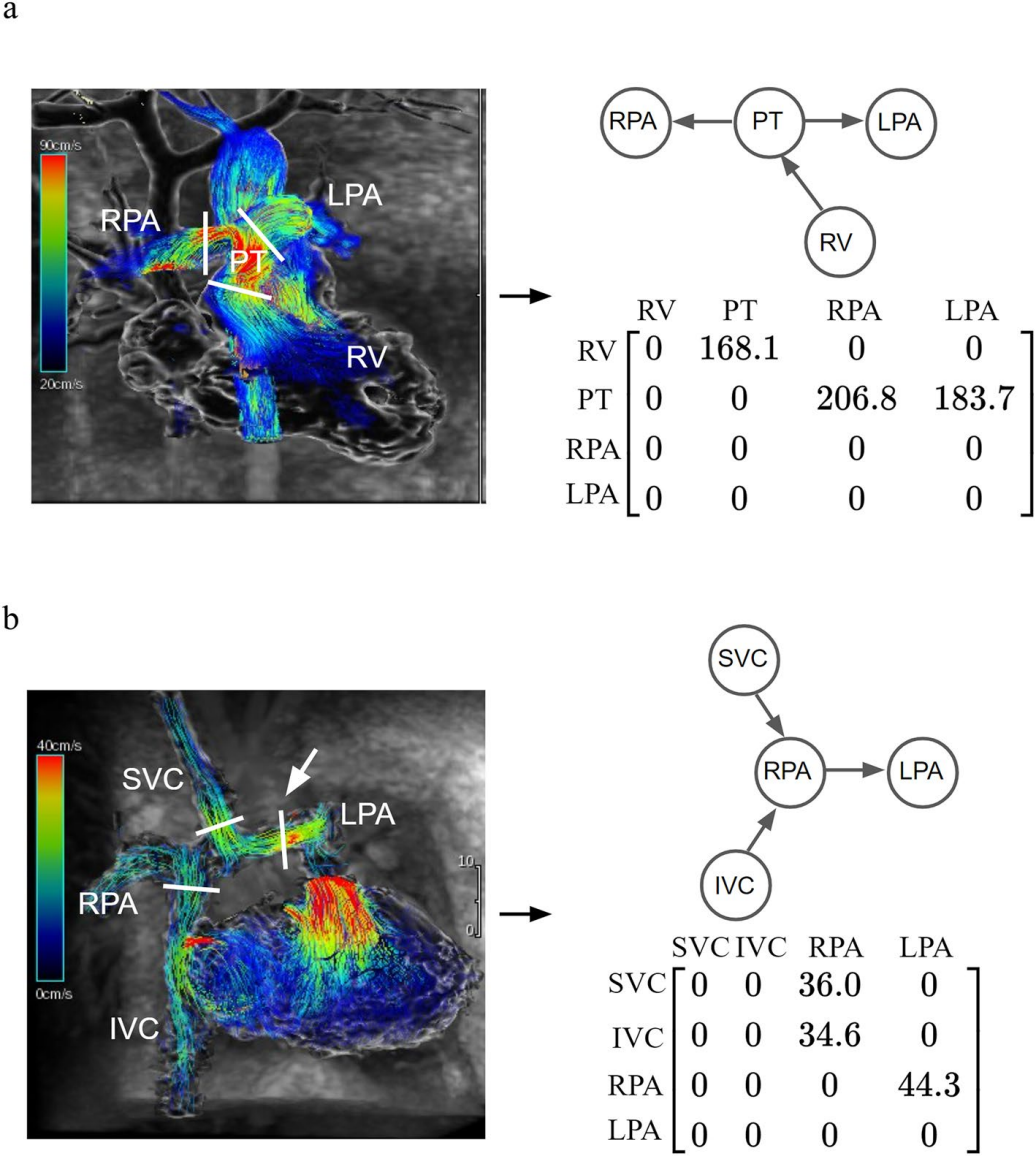
Construction of the weighted adjacency matrices of (**a**) surgically corrected d-TGA and (**b**) Fontan circulation. Blood flow visualization with velocity color-coded streamlines on 4D flow MRI. The short white lines are the boundaries between adjacent structures where the peak velocities (in cm/s) are measured. Similar to totally repaired TOF, the peak velocities were measured between the RV, PT, RPA, and LPA in the case of surgically corrected d-TGA. For the Fontan circulation, the PT was separated from the RPA and LPA; the SVC and IVC were connected to RPA. The peak velocities were measured between the SVC, RPA, IVC, and LPA. The white arrow indicates the boundary between RPA and LPA, as well as the location of native pulmonary bifurcation. *4D flow MRI* four-dimensional flow magnetic resonance imaging, *IVC* inferior vena cava, *LPA* left pulmonary artery, *PT* pulmonary trunk, *RPA* right pulmonary artery, *RV* right ventricle, *SVC* superior vena cava, *TGA* transposition of the great arteries, *TOF* tetralogy of Fallot.

The data of three patients who underwent 4D flow MRI and had three different conditions—totally repaired TOF, surgically corrected d-TGA, and Fontan circulation—were included. The process of constructing the weighted adjacency matrix for totally repaired TOF based on the results of 4D flow MRI is shown in Fig. 3. Regarding flow measurements, we usually only focus on a specific portion of the heart in clinical practice. Therefore, a submatrix of the binary adjacency matrix of the whole heart was selected to create the weighted adjacency matrix. In this case, we were interested in the peak velocities of blood flows between the RV, PT, RPA, and LPA. Initially, the peak velocities were measured at the boundaries of the spaces on 4D flow MRI (pulmonary valve and the origins of the RPA and LPA, the short white lines in Fig. 3) and were used as weights in the weighted adjacency matrix. Then, the submatrix including the vertices of interest was derived from the binary adjacency matrix of totally repaired TOF. After the weights and corresponding elements of the submatrix were combined, the weighted adjacency matrix of peak velocity was obtained. In the same way, the weighted adjacency matrices of surgically corrected d-TGA and Fontan circulation were constructed (Fig. 4).

## Discussion

This study provided a representation of CHDs through graphs, binary and weighted adjacency matrices by using graph theory. We generated graph representations of the normal heart, TOF, and TGA. Based on the differences between the graph of the normal heart and that of CHDs, CHDs can be classified into five categories: (1) increased vertices; (2) decreased vertices; (3) increased edges; (4) decreased edges; and (5) abnormal connections. Examples for the five categories of binary adjacency matrices are shown in Table 1. The procedures to treat CHDs is also related to the addition and subtraction of these vertices and edges, as well as the correction of abnormal connections. Manifestations of the common surgical procedures in the binary adjacency matrices are shown in Table 2. For the CHDs and procedures associated with changes in vertices, we increased the dimensions of the adjacency matrices for the category of increased vertices while keeping the dimension unchanged for the category of decreased vertices. The reason for this is that a vertex is a chamber or a vascular structure with a real space and can connect to other vertices. Therefore, an additional vertex requires more dimension in the adjacency matrix to express the possible connections with all the other vertices. By contrast, the absence of a vertex can be expressed by eliminating all the edges connected to the vertex. Thus, isolating the vertex from the other vertices is equivalent to removal of the vertex from the graph in our cases.

**Table 1:** Manifestations of the CHDs in the binary adjacency matrices.

CHDs	Manifestations of the CHDs in the binary adjacency matrices A = [*a*_*ij*_]
Increased vertices	
PDA	*v*_25_ = PDA, *a*_18,25_ = 1, *a*_25,5_ = 1
MAPCA	*v*_25_ = MAPCA, *a*_18,25_ = 1, *a*_25,8_ = 1 or *a*_25,9_ = 1
PLSVC	*v*_25_ = PLSVC, *a*_25,3_ = 1
Decreased vertices	
Isolated unilateral absence of pulmonary artery	*a*_5,6_ = 0, *a*_6,8_ = 0 (right) or *a*_5,7_ = 0, *a*_7,9_ = 0 (left)
Increased edges	
ASD	*a*_3,14_ = 1 or *a*_14,3_ = 1
VSD	*a*_4,15_ = 1 or *a*_15,4_ = 1
Aortopulmonary septal defect	*a*_5,16_ = 1 or *a*_16,5_ = 1
Decreased edges	
Tricuspid atresia	*a*_3,4_ = 0
Pulmonary atresia	*a*_4,5_ = 0
Abnormal connections	
Anomalous pulmonary venous return	For an *i* ∈ {10, 11, 12, 13} (pulmonary veins), there exists a *j* ≠ 14 (*v*_14_ = LA) such that *a*_*ij*_ = 1
D-TGA	*a*_3,4_ = 1, *a*_3,15_ = 0, *a*_14,15_ = 1, *a*_14,4_ = 0 and *a*_4,5_ = 0, *a*_4,16_ = 1, *a*_15,16_ = 0, *a*_15,5_ = 1 (interchanging 5th column (PT) and 16th column (AAo) of the matrix of normal heart)
L-TGA	*a*_3,4_ = 0, *a*_3,15_ = 1, *a*_14,15_ = 0, *a*_14,4_ = 1 (interchanging 4th column (RV) and 15th column (LV) of normal heart) and *a*_4,5_ = 0, *a*_4,16_ = 1, *a*_15,16_ = 0, *a*_15,5_ = 1 (interchanging 5th column (PT) and 16th column (AAo) of the matrix of normal heart)
Double outlet right ventricle	*a*_4,5_ = 1, *a*_4,16_ = 1, *a*_15,16_ = 0, *a*_15,5_ = 0
Truncus arteriosus	*v*_16_ = truncus arteriosus, *a*_4,5_ = 0, *a*_4,16_ = 1, *a*_15,16_ = 1, *a*_15,5_ = 0, *a*_16,5_ = 1

*AAo* ascending aorta, *ASD* atrial septal defect, *CHD* congenital heart disease, *LA* left atrium, *LV* left ventricle, *MAPCA* major aortopulmonary collateral artery, *PDA* patent ductus arteriosus, *PLSVC* persistent left superior vena cava, *PT* pulmonary trunk, *RV* right ventricle, *TGA* transposition of the great arteries, *VSD* ventricular septal defect.

**Table 2:** Manifestations of the procedures in the binary adjacency matrices.

Procedures	Manifestations of the postoperative CHDs in the binary adjacency matrices B = [*b*_*ij*_]
Right mBT shunt	*v*_25_ = mBT shunt, *b*_23,25_ = 1, *b*_25,6_ = 1
Left mBT shunt	*v*_25_ = mBT shunt, *b*_21,25_ = 1, *b*_25,7_ = 1
Norwood procedure with a right mBT shunt	*b*_5,6_ = 0, *b*_5,7_ = 0, *b*_5,16_ = 1; *v*_25_ = mBT shunt, *b*_23,25_ = 1, *b*_25,6_ = 1, *b*_6,7_ = 1
Bidirectional Glenn shunt	*b*_1,3_ = 0, *b*_1,6_ = 1
Fontan procedure	*b*_2,3_ = 0, *b*_2,6_ = 1
Arterial switch procedure	Interchanging 5th column (PT) and 16th column (AAo) of the matrix

*AAo* ascending aorta, *CHD* congenital heart disease, *mBT shunt* modified Blalock–Taussig shunt, *PT* pulmonary trunk.

Our methods for defining the vertices and edges of the graph are similar to those used in studies on graph theory applications in the human heart^5–8^. However, our models are more detailed and practical than those used in previous studies. For example, our model comprised four vertices to represent four pulmonary veins. This design is crucial for describing anomalous pulmonary venous return but has been rarely used in other studies. In addition, most previous models have only included the cardiovascular structures from the vena cavae to the aorta. By contrast, we included the structures from the vena cavae to the branches of the aorta because the branches include the innominate artery, carotid arteries, and subclavian arteries, which also play a role in CHDs and are often associated with additional blood supply to the lungs, such as mBT shunt and systemic-to-pulmonary collateral arteries. Moreover, we added the vertex of SCTO to complete the circuit of the entire circulatory system. Abnormalities in the connections between systemic arterial supply, SCTO, and systemic venous return are usually associated with cardiovascular diseases in adults such as atherosclerotic occlusive diseases, arterial dissections, deep vein thrombosis, and SVC syndrome. The aim of this study was not to establish the standard graph representation of the human heart but to propose a feasible model. Various graph representations can be adopted depending on the purpose. For instance, if information on more detailed structures, such as coronary arteries, segments of the aorta, and pulmonary arteries, is required, more vertices can be added into the graph. Moreover, the graph representations can include dynamic changes obtained from imaging modalities such echocardiography and cardiac MRI. Finite-state machine may be used to describe the dynamic changes of blood flow, chamber and vessels in the cardiac cycle^19,20^.

In addition to the human heart, graph theory has been applied to other systems and organs in the human body. One of the most important applications of graph theory is the connectome in neuroscience. Since the coining of the term connectome in 2005, the number of related studies has markedly increased, with more than 1000 publications per year over the past few years^21^. Graph theoretical analysis of complex brain networks reveals that the human connectome can be a diagnostic marker of several neurodegenerative and psychiatric diseases^22^. Graph theory is also used to build models of brain vessels, the hepatic portal system, the glomerular capillary network, and the lymphatic system^23–29^.

The increasing survivorship of patients with CHD increases the prevalence of CHD, and as a result, physicians without having training in CHD may need to manage these patients^30^. Graph representation simplifies the complex cardiovascular structures and makes them easier to understand for the physicians and patients. Furthermore, according to previous graph theory-based studies, we propose some applications of our CHD model.

### Artificial intelligence in CHD

If there is an artificial intelligence model capable of segmenting the cardiovascular structures in CHDs on the imaging, we can obtain the boundaries of these structures and detect the blood flow between them. By doing so, we can establish the connections between these structures and construct the graphs of the hearts. By comparing these graphs with the established graphs of CHDs in this study, we can obtain the diagnoses for the congenital heart diseases of the hearts. For example, if the morphologic RA, RV, LA, LV, AAo and PT can be automatically identified and segmented on MRI, atrioventricular and ventriculoarterial connections can be established using 4D flow MRI. If blood flow is detected from the RV to the AAo and from the LV to the PT, the diagnosis of TGA can be made (Fig. 1c). Furthermore, through 4D flow MRI and appropriate segmentation of cardiovascular structures, hemodynamic abnormalities such as regurgitation and increased velocity due to vascular stenosis can be automatically detected. Although the automatic segmentation and classification of CHD in medical imaging are challenging due to the complex cardiovascular structures involved, with advances in machine learning techniques, many feasible models have been proposed^31–33^.

For automatic segmentation, graph matching, which is a graph theory-based technique, has been adopted to improve the performance of deep learning models. Graph matching in a previous study involved the construction of a graph library to describe all possible connections between great vessels^32^. Various graph representations and categories of CHDs demonstrated in the present study can be used to establish a comprehensive graph library and can facilitate the development of deep learning in CHD.

### Connectome of the heart

Similar to the application of the connectome in the neurological disorders, graph representations of the heart can be applied to heart diseases^34^. In this study, we demonstrated that the changes in the numbers of vertices, edges, and connections were associated with the structural or hemodynamic abnormalities of the heart. In addition, imaging modalities such as echocardiography and cardiac MRI, similar to electroencephalography, diffusion tensor imaging, and functional MRI in neuroscience, can be used to construct weighted adjacency matrices, which can store the hemodynamic parameters of the heart in the matrices and enable further analysis to find unknown relationships between the matrices and heart diseases^35–37^.

### Finding unknown types, pathophysiology, and treatments of CHDs

The number of all possible directed graphs with 24 vertices is 2^(24×23)^–1.47 × 10^166^, which is considerably more than the types of CHDs currently known. This observation implies that some cardiovascular structures of the possible directed graphs have not yet been discovered or reported, while some cardiovascular structures cannot exist due to certain reasons. We can find unknown types of CHDs through the following steps: (1) given 24 vertices, as defined above, generate all possible directed graphs; (2) exclude the known CHDs based on our knowledge and literal searches on databases such as PubMed and Google Scholar; (3) the remaining non-excluded directed graphs are the possible unknown cardiovascular structures of CHDs. For each possible unknown cardiovascular structure, we can use our knowledge of physiology and embryology to determine the existence of this cardiac structure. A well-known rule for patients with CHDs to survive is “there exists a lung such that a path from the systemic venous return to the lung and a path from the lung to the systemic arterial supply”, which we can see in the patients with extreme TOF and d-TGA^14^. For instance, if a patient has d-TGA without a VSD, there is no blood flow between the RV and the PT. The deoxygenated blood from the systemic venous return (SVC, IVC) flows into the RV and is then pumped directly into the systemic arterial supply (AAo) without undergoing gas exchange in the lungs (Fig. 1c). The patient cannot survive due to a lack of oxygenated blood in the systemic circulation. In addition, other unknown factors may also affect survival of CHD. Therefore, scrutinizing all possible cardiovascular structures by using our model, we may find unknown CHDs and unknown rules associated with the existence of CHDs, that is, (cardiovascular structures of all possible directed graphs with the given vertices) − (known cardiovascular structures) − (abnormal cardiovascular structures incompatible with life because of the known rules) = (abnormal cardiovascular structures compatible with life but not reported yet) + (abnormal cardiovascular structures incompatible with life because of unknown rules). For example, congenital anomalies such as congenital total absence of SVC and congenital aortocaval fistula to the SVC are extremely rare anomalies and have never been recorded in some institutes^38,39^. However, the existence of the anomalies can be predicted using our model. Furthermore, the procedures for treatment of CHDs such as mBT shunt, Norwood procedure, bidirectional Glenn shunt and Fontan procedure can also be derived from our model. The unknown treatments of CHDs may be discovered in all possible directed graphs with the given vertices.

The proposed graph representations of CHDs have some limitations. They cannot describe abnormal spatial relationships between cardiovascular structures such as the situs inversus, L-loop, overriding aorta, right aortic arch, aberrant subclavian artery, retroaortic left innominate vein, and anomalous course of coronary arteries. Some spatial relationships may be represented by adding new vertices based on embryology^40,41^. Moreover, a binary adjacency matrix cannot describe two edges with the same origin vertex, destination vertex and direction. Further research is needed to find the more general graph models to describe CHDs.

In conclusion, graph theory can be used to represent CHDs, which may be helpful for developing artificial intelligence and conducting future research on CHD.

## Data Availability

The datasets used and/or analyzed during the current study available from the corresponding author on reasonable request.

## References

[1] Lapierre C (2010). Segmental approach to imaging of congenital heart disease. Radiographics.

[2] Gladman G, McCrindle BW, Williams WG, Freedom RM, Benson LN (1997). The modified Blalock-Taussig shunt: Clinical impact and morbidity in Fallot's tetralogy in the current era. J. Thorac. Cardiovasc. Surg..

[3] Norwood WI, Jacobs ML, Murphy JD (1992). Fontan procedure for hypoplastic left heart syndrome. Ann. Thorac. Surg..

[4] Pavlopoulos GA (2011). Using graph theory to analyze biological networks. BioData Mining.

[5] Shokry M, Aly RE (2013). Topological properties on graph vs medical application in human heart. Int. J. Appl. Math..

[6] Basavaprasad B, Ravindra SH (2014). A graph theoretical network model on human heart. Int. J. Appl. Eng. Res..

[7] Nawar AS, El Atik AEFA (2019). A model of a human heart via graph nano topological spaces. Int. J. Biomath..

[8] Abeyrathne R, Lanel G (2021). A study on graph theory properties in human blood circulatory system. Int. J. Sci. Res. Publ..

[9] Othman HA (2022). Pathless directed topology in connection to the circulation of blood in the heart of human body. AIMS Math..

[10] El Atik AEFA (2022). A topological approach of a human heart via nano pre-ideality. Thai J. Math..

[11] Apitz C, Webb GD, Redington AN (2009). Tetralogy of fallot. The Lancet.

[12] Coelho E (1961). Tetralogy of Fallot: Angiocardiographic, electrocardiographic, vectorcardiographic and hemodynamic studies of the Fallot-type complex. Am. J. Cardiol..

[13] Van Praagh R (1977). Terminology of congenital heart disease. Glossary and commentary. Circulation.

[14] Warnes CA (2006). Transposition of the great arteries. Circulation.

[15] Di Salvo G (2018). Imaging the adult with congenital heart disease: A multimodality imaging approach—Position paper from the EACVI. Eur. Heart J.-Cardiovasc. Imaging.

[16] Sachdeva S, Gupta SK (2020). Imaging modalities in congenital heart disease. Indian J. Pediatr..

[17] Azarine A (2019). Four-dimensional flow MRI: Principles and cardiovascular applications. Radiographics.

[18] Jacobs K (2021). Hemodynamic assessment of structural heart disease using 4D flow MRI: How we do it. Am. J. Roentgenol..

[19] Zhang Z, Xia C, Fu J, Chen Z (2022). Initial-state observability of mealy-based finite-state machine with nondeterministic output functions. IEEE Trans. Syst. Man Cybern. Syst..

[20] Zhang Z, Shu S, Xia C (2021). Networked opacity for finite state machine with bounded communication delays. Inf. Sci..

[21] Veldhuizen MG (2022). Future directions for chemosensory connectomes: Best practices and specific challenges. Front. Syst. Neurosci..

[22] Bullmore E, Sporns O (2009). Complex brain networks: Graph theoretical analysis of structural and functional systems. Nat. Rev. Neurosci..

[23] Paetzold, J. C. *et al. Thirty-fifth Conference on Neural Information Processing Systems Datasets and Benchmarks Track (Round 2).*

[24] Reichold J (2009). Vascular graph model to simulate the cerebral blood flow in realistic vascular networks. J. Cereb. Blood Flow Metab..

[25] El Azab MS, Shokry M, Emad Aly R (2022). A new view of special types of subgraphs with applications on circulation of hepatic portal system. Inf. Sci. Lett..

[26] Wahl EM, Daniels FH, Leonard E, Levinthal C, Cortell S (1984). A graph theory model of the glomerular capillary network and its development. Microvasc. Res..

[27] Savinkov R (2020). Graph theory for modeling and analysis of the human lymphatic system. Mathematics.

[28] Tretyakova R, Savinkov R, Lobov G, Bocharov G (2017). Developing computational geometry and network graph models of human lymphatic system. Computation.

[29] Mozokhina A, Savinkov R (2020). Mathematical modelling of the structure and function of the lymphatic system. Mathematics.

[30] Hoffman JIE, Kaplan S, Liberthson RR (2004). Prevalence of congenital heart disease. Am. Heart J..

[31] Pace, D. F. *et al. International Conference on Medical Image Computing and Computer-Assisted Intervention* 80–88 (Springer).

[32] Xu, X. *et al.**International Conference on Medical Image Computing and Computer-Assisted Intervention* 477–485 (Springer).

[33] Xu, X. *et al.**International Conference on Medical Image Computing and Computer-Assisted Intervention* 77–87 (Springer).

[34] Stam CJ, Jones BF, Nolte G, Breakspear M, Scheltens P (2007). Small-world networks and functional connectivity in Alzheimer's disease. Cereb. Cortex.

[35] Vecchio F, Miraglia F, MariaRossini P (2017). Connectome: Graph theory application in functional brain network architecture. Clin. Neurophysiol. Pract..

[36] Iturria-Medina Y (2007). Characterizing brain anatomical connections using diffusion weighted MRI and graph theory. Neuroimage.

[37] Salvador R (2005). Neurophysiological architecture of functional magnetic resonance images of human brain. Cereb. Cortex.

[38] Oliveira JD, Martins I (2019). Congenital systemic venous return anomalies to the right atrium review. Insights Imaging.

[39] Soler P, Mehta AV, Garcia OL, Kaiser G, Tamer D (1981). Congenital systemic arteriovenous fistula between the descending aorta, azygos vein, and superior vena cava. Chest.

[40] Tong E, Rizvi T, Hagspiel KD (2015). Complex aortic arch anomaly: Right aortic arch with aberrant left subclavian artery, fenestrated proximal right and duplicated proximal left vertebral arteries-CT angiography findings and review of the literature. Neuroradiol. J..

[41] Gerlis LM, Ho S (1989). Anomalous subaortic position of the brachiocephalic (innominate) vein: A review of published reports and report of three new cases. Heart.

